# Attachment on mortar surfaces by cyanobacterium *Gloeocapsa* PCC 73106 and sequestration of CO_2_ by microbially induced calcium carbonate

**DOI:** 10.1002/mbo3.1243

**Published:** 2021-10-14

**Authors:** Tingting Zhu, Mohamed L. Merroun, George Arhonditsis, Maria Dittrich

**Affiliations:** ^1^ Biogeochemistry Laboratory Department of Physical and Environmental Sciences University of Toronto Scarborough Toronto ON Canada; ^2^ Department of Microbiology University of Granada Granada Spain; ^3^ Ecological Modelling Laboratory Department of Physical and Environmental Sciences University of Toronto Scarborough Toronto Ontario Canada; ^4^ Present address: Department of Geography, Geomatics and Environment Department of Mathematical and Computational Sciences University of Toronto Mississauga 3359 Mississauga Rd Mississauga Ontario L5L 1C6 Canada

**Keywords:** cyanobacterium *Gloe*. PCC 73106, extracellular polymeric substances, microbially induced carbonate precipitation, mortar durability

## Abstract

Cyanobacterial carbonate precipitation induced by cells and extracellular polymeric substances (EPS) enhances mortar durability. The percentage of cell/EPS attachment regulates the effectiveness of the mortar restoration. This study investigates the cell coverage on mortar and microbially induced carbonate precipitation. Statistical analysis of results from scanning electron and fluorescence microscopy shows that the cell coverage was higher in the presence of UV‐killed cells than living cells. Cells are preferably attached to cement paste than sand grains, with a difference of one order of magnitude. The energy‐dispersive X‐ray spectroscopy analyses and Raman mapping suggest cyanobacteria used atmospheric CO_2_ to precipitate carbonates.

## Graphical abstract

Cyanobacterial carbonate precipitation induced by cyanobacterium *Gloeocapsa* enhances mortar durability. Cells are preferably attached to cement paste than sand grains and use atmospheric CO_2_ to precipitate carbonates. The cell coverage is higher in the presence of UV‐killed cells than living cells, providing insights for microbial mortar restoration.

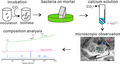

## 1 INTRODUCTION

Microbially induced carbonate precipitation (MICP) has demonstrated its potential in enhancing the durability of building materials, such as mortars and concrete (Achal & Mukherjee, 2015; Achal et al., 2011; Lors et al., 2017; Zhu et al., 2017). By depositing carbonates in pores, micro‐cracks, or on the mortar surface, MICP decreases the penetration of aggressive chemicals such as chlorides, sulfates, and acids that would corrode steels (Bansal et al., 2016). As a result, it reduces the regular maintenance and repair of constructions that are highly labor dependent and costly (Toncheva‐Panova et al., 2010). Bio‐deposition on mortar is an effective way to enhance its durability since the degradation mostly starts from the cracked surface (Stephen & Gettu, 2020).

A variety of microbes such as ureolytic bacteria, myxobacteria, and cyanobacteria have been applied to building materials (Zhu & Dittrich, 2016). Through ureolysis or ammonification, ureolytic bacteria and myxobacteria increase the pH and produce CO_2_ using urea or amino acids to promote the carbonate precipitation process (Gonzalez‐Munoz et al., 2010). However, ammonia (NH_3_), a common air pollutant, is produced in both processes (Mitchell et al., 2013). Cyanobacteria, on the contrary, only require easily prepared nutrient solutions and do not generate side products during carbonate precipitation. Therefore, microbially induced carbonate precipitation using cyanobacteria is less expensive and more sustainable (Toncheva‐Panova et al., 2010). *Gloeocapsa* PCC 73106 (*Gloe*. PCC 73106) is biofilm‐forming bacteria that were found to live on building materials such as limestone (Carlos Rodriguez‐Navarro et al., 2012). For all these reasons, the present study investigates the potential of carbonate precipitation by cyanobacteria *Gloe*. PCC 73106 on building material mortar. Since the pH around mortar surfaces is already high, the conditions are conducive to carbonate precipitation and the pH increase by cyanobacterial photosynthesis is not expected to be an important contributor (Zhu et al., 2017). As such, the efficiency of both living and UV‐killed *Gloe*. PCC 73106 to mediate carbonate precipitation is investigated in this study.

One critical question that deserves further research is the attachment of cells on mortar and their detachment during the MICP application. Bacteria with negative surface charge are easily retained on positively charged or neutral surfaces, thereby favoring adhesion, and ultimately restoration of the substrates by creating a supersaturated microenvironment for heterogeneous crystallization (De Muynck et al., 2010). Several concerns may arise if the attachment of cells on mortar surfaces is low, reversible, or incomplete. For example, cells would spread to the environment and result in uncontrolled growth. Another concern is that the protection will be less effective due to the low coverage of cells on substrates (Surabhi & Arnepalli, 2020). It is also imperative for cells to attach to the right location, such as on porous areas or in micro‐cracks for successful protection.

The process of MICP requires sources of carbon, nutrients, and calcium, but the impact of the type and amount of carbon sources, including organic carbon, bicarbonate, carbonate, or CO_2_ on MICP has been understudied. For example, organic carbon is used by heterotrophs to generate bicarbonate or CO_2_ for the following carbonate precipitation. *Sporosarcina pasteurii*, a ureolytic bacteria strain, hydrolyzes urea to produce CO_2_, contributing to the gradual precipitation of calcium carbonate (Abdel‐Aleem et al., 2019; Bang et al., 2001). Similarly, myxobacteria produce CO_2_ through ammonification to aid carbonate precipitation (C. Rodriguez‐Navarro et al., 2003). Inorganic carbon such as CO_2_ or HCO_3_^−^ can be used by photosynthetic cyanobacteria to increase pH, thereby creating a microenvironment favoring carbonate precipitation (Badger & Price, 2003; Riding, 2006). Since the types and amount of carbon sources added in the MICP applications impact the environment, the selection of carbon sources is essential. Atmospheric CO_2_ can be sequestrated by cyanobacteria in stabilizing the mine tailings (McCutcheon et al., 2016), but no one has investigated the CO_2_ sequestration by cyanobacteria on mortar. Previously, we reported that cyanobacterial carbonate precipitation (CCP) utilizing HCO_3_^−^ improved the mortar performance (Zhu et al., 2015, 2018). The present study investigates CCP through atmospheric CO_2_ on mortar, which has the potential to be a sustainable carbon source for carbonate precipitation. The latter process can also contribute to greenhouse‐effect mitigation by decreasing the atmospheric CO_2_.

In this study, one of our primary objectives is the comparison of the attachment and detachment of living and UV‐killed *Gloe*. PCC 73106 with and without the amendment of calcium ions on mortar surfaces. The coverage and cell density on mortar surfaces were monitored by fluorescence microscope and scanning electron microscope (SEM). The early stage of calcium carbonate precipitation was investigated in an open system, where atmospheric CO_2_ was the sole carbon source. The composition of the precipitates, cells, and mortar was analyzed using energy‐dispersive spectroscopy (EDS) and Raman spectroscopy. Both microscopy and spectroscopy showed that, in comparison with living cells, UV‐killed *Gloe*. PCC 73106 contributed to higher coverage of cells on mortar surfaces. A higher percentage of living‐cell attachment was achieved in the presence of calcium ions than without. Both living and UV‐killed cells led to calcium carbonate precipitation, including aragonite and vaterite.

## 2 METHODS AND MATERIALS

### 2.1 Bacterial strain and cultivation conditions

Experiments were performed with *Gloe*. PCC 73106 strain, a biofilm‐forming bacterial strain received from Pasteur Culture Collection. Culture was grown in BG‐11 medium comprised of NaNO_3_ 17.6 mM, K_2_HPO_4_ 0.23 mM, MgSO_4_·7H_2_O 0.3 mM, CaCl_2_·2H_2_O 0.24 mM, citric acid·H_2_O 0.031 mM, ferric ammonium citrate 0.021 mM, Na_2_EDTA·2H_2_O 0.0027 mM, Na_2_CO_3_ 0.19 mM and BG‐11 trace metal H_3_BO_3_ 46 mM, MnCl_2_·4H_2_O 9 mM, ZnSO_4_·7H_2_O 0.77 mM, Na_2_MoO_4_·2H_2_O 1.6 mM, CuSO_4_·5H_2_O 0.3 mM, Co(NO_3_)_2_·6H_2_O 0.17 mM. Batch cultures were constantly shaken at 110 rpm at room temperature 22 ± 1°C with a white light lamp at an intensity of 20 µE m^−2^ S^−1^ (HOBO Micro Station H21‐002 light sensor).

**FIGURE 1:**
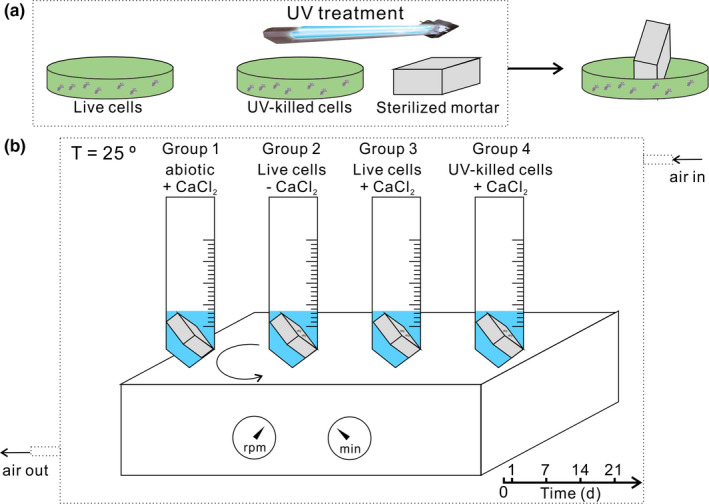
Experimental setup. (a) Pretreatment of mortar. (b) Experimental details

After reaching the stationary growth stage (14 days), cells were collected by centrifuging at 3480 g for 15 min. This step was followed by three cycles of cell wash in a sterilized 0.1 M NaNO_3_ solution through centrifuging. After the last wash, cells were resuspended in the deionized water. To ensure the equal amount of the cells used for different conditions (groups 2, 3, and 4), the cell suspension was equally divided into three parts. One‐third of the cell suspension was exposed to UV light for 1 hour and was killed, while the rest was kept for the experiments (Figure 1a). The UV‐killed cells were reinoculated in BG‐11 medium in triplicates, and none of them showed the growth of cells.

### 2.2 Experiments on mortar with living and UV‐killed cells

Mortar cubes with a size of 30 × 30 × 15 mm were cast using SAKRETE^®^ Sand Mix in a customized mold. The sand‐to‐cement ratio was 11:4. They were demolded after 24 hours and cured for 28 days under a humid atmosphere (90% R.H., 20°C). Each cube was then cut into 8 pieces with a size of 15 × 15 × 6 mm. The top surface (15 × 15 mm) of each cube was polished using 200, 400, 800, and 1200 grit polishing papers. The polished cubes were washed with acetone in a sonication bath for 5 min and followed by a wash in deionized water.

Just before the experiments, mortar cubes were exposed to UV light for 1 hour to kill the native microorganisms and distinguish the contribution of the cyanobacterial strain used in the carbonate precipitation (Figure 1a). They were then divided into four groups, of which group 1 was treated abiotically in sterilized deionized water, groups 2 and 3 were immersed in living bacterial suspension (10^9^ cells/ml), and group 4 was immersed in UV‐killed bacterial suspension (10^9^ cells/ml). The immersion took place in Petri dishes, and only the bottom half of the large surface (15 × 15 mm) cube was in the cell suspension (Figure 1a). After this step, half of the studied surface was covered with bacteria. Cubes were removed from the suspension after 30 min and held in a tilted position for 30 s to allow the excess water to drop off. The wet cubes were transferred to falcon tubes (Figure 1b), filled with 15 mL calcium chloride solution (300 mM) in groups 1, 3, and 4, and 15 mL sterilized deionized water without calcium chloride in group 2. The experiments were carried out in an incubator with temperature set to 25 ºC and circulated with compressed air containing 0.04% CO_2_ to avoid the varying concentration of CO_2_ due to human activities in the laboratory. Samples in falcon tubes were constantly shaken under 60 rpm with a light intensity of 20 µE m^−2^ S^−1^ and were examined after 1, 7, 14, and 21 d. All experiments were conducted in triplicates.

### 2.3 Microscopy and spectroscopy

The bulk solution initially contained zero cells. During the experiments, the bulk solution was filtered with a 0.2 μm polycarbonate membrane filter to count the cells that were detached from the concrete cubes and spread to the bulk solution. Cells spread to the bulk solution were counted using an OLYMPUS IX51 fluorescence microscope with a TRITC‐A‐OMF filter (exciter, 542/20‐25 nm; emitter, 620/52‐25 nm; dichroic beam splitter, 570 nm). Aliquots at 1, 7, 14, and 21 d in experiments were filtered through a 0.2 µm black polycarbonate Nuclepore filter and examined under an epifluorescence microscope. One hundred random areas with a size of 5701 μm^2^ on the filter were captured per sample, and the cells were counted. The size of the effective filter area was 490.87 mm^2^, and the volume of the filtered aliquots was 2 ml. The cell concentration (cells/ml) in the bulk solution was then calculated using *N*_sum_/(100*5701*10^−6^)*490.87/2, in which *N*_sum_ is the total number of cells counted in one hundred random areas.

**FIGURE 2:**
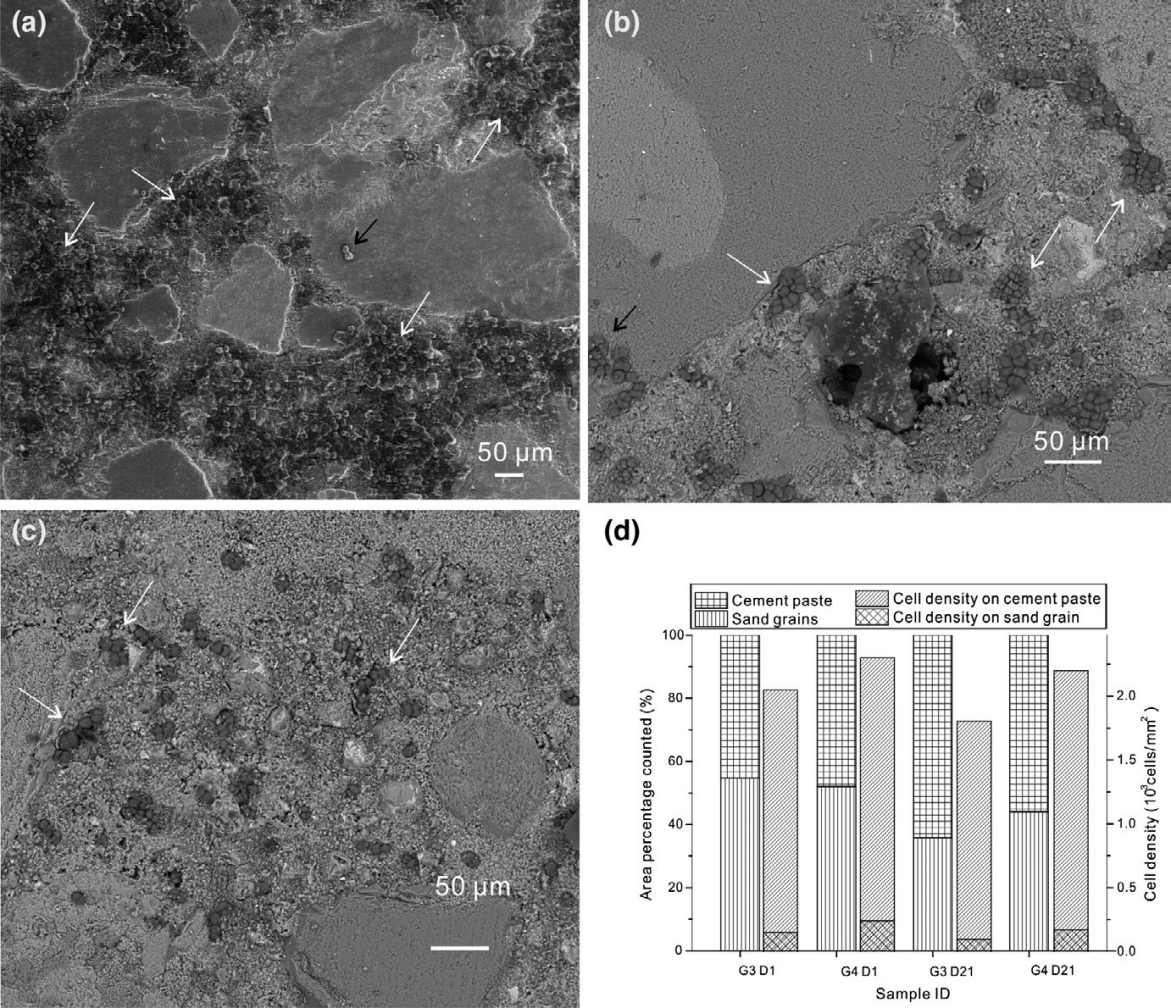
SEM images showing the differential attachment of cells (white arrows) to cement paste and sand grains on mortar surfaces on (a) days 0, (b) 1, and (c) 21 in group 3. (d) The percentage of cement paste and sand grains on the counted area, and the cell density on these two substrates in group 3 and group 4 on days 0 and 21

**FIGURE A1:**
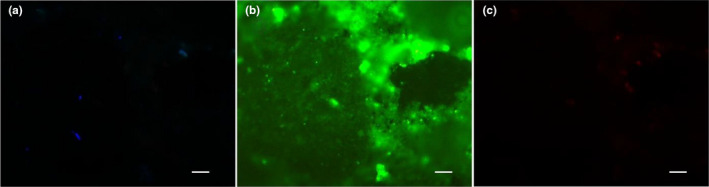
Autofluorescence of the concrete surface. (a) blue channel with DAPI filter set (exciter, 352–402 nm; emitter, 417–477 nm), (b) green channel with FITC filter set (exciter, 467–498 nm; emitter, 513–556 nm), and (c) red channel with TRITC filter (exciter, 546 nm, emitter, 575–640 nm). (scale bar is 50 µm).

**FIGURE A2:**
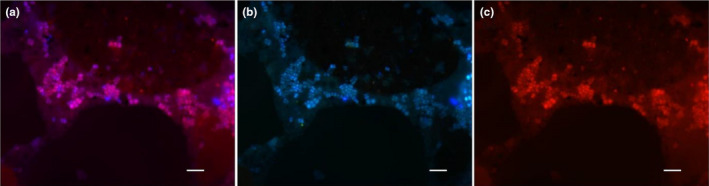
Autofluorescence of cyanobacteria PCC 73106 cells on day 7. (a) composite of the blue channel with DAPI filter set (exciter, 352–402 nm; emitter, 417–477 nm) and red channel with TRITC filter (exciter, 546 nm, emitter, 575–640 nm), (b) blue channel, (c) red channel. (scale bar is 50 µm).

**FIGURE A3:**
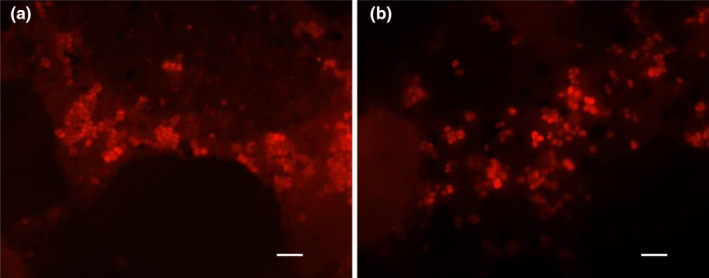
Autofluorescence of PCC 73106 on day 7. (a) living cells, b) UV‐killed cells. (scale bar is 50 µm).

**FIGURE A4:**
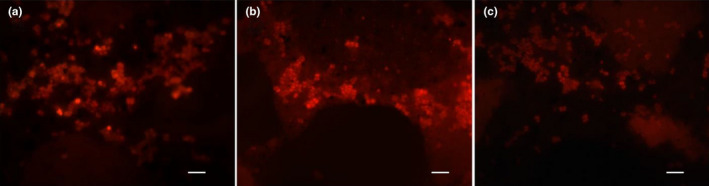
Autofluorescence of PCC 73106 living cells on a) day 0, b) day 7, c) day 14. All of the images were taken under the same settings. (scale bar is 50 µm).

Cells attached to mortar surfaces were observed using a Zeiss Axioplan 2 fluorescence microscope with a TRITC filter (exciter, 546 nm, emitter, 575–640 nm, and dichroic beam splitter, 560 nm) that is similar to the filter set used by Neu et al. (2004). Mortar treated with living and UV‐killed cells were examined on days 0, 1, 7, 14, and 21 days. Since cells have autofluorescence, they exhibited red in the captured image. Additional information regarding the autofluorescence of concrete, live, and UV‐killed cells is provided in the Appendices (Figures A1, A2, A3, A4). The cell coverage on the mortar surface was automatically calculated using ImageJ by transferring images to an 8‐bit greyscale (Figure 2a and b) and adjusting the threshold. A constant threshold was used for all images. At least 10 images with a frame size of 631 × 500 μm were analyzed for each sample.

The attached cells on mortar surfaces and cell morphology were observed under Supra 40VP field emission scanning electron microscopy and CAMECA SX100 environmental scanning electron microscopy (FESEM and ESEM at CIC, University of Granada, Spain), both coupled with EDS microanalysis. The EDS detector is a 50 mm^2^ silicon drift detector XMAX enabling detection of elements with Z≥ 4(Be) and high‐count rates. A quantitative proxy of the elements using virtual standards of the microanalysis system, Aztec 2.2, with normalization of 100 was conducted. PbZAF model was used in EDS quantification. The operating conditions were set to accelerating voltage of 15 kV and live time acquisition of 30 s. The spatial resolution of EDS microanalysis depends upon the beam size, nature of the matrix analyzed and the beam energy used. In this study, the spatial resolution is lower than 1 µm. Samples for FE‐SEM and ESEM were carbon‐coated; therefore, the EDS results did not include carbon. The elemental composites of 80 spots on the mortar surface, cells, EPS, and precipitates were analyzed by EDS. Cells attached to the mortar surface were counted manually. The total area counted was 1.5×10^6^ μm^2^, and the total cells counted were between 200 and 500 for each cube.

Raman spectroscopy analyses for samples were carried out under the NTEGRA Spectra system from NT‐MDT (Russia) equipped with an upright optical microscope. Raman spectra were acquired using 532 nm excitation wavelength at ca. 4 mW. The power intensity was adjusted with an ND filter. An acquisition period of 0.5 s repeated by 300 was adopted for all of the spectra. Confocal 2D Raman mapping was obtained on a mortar surface treated with living cells. The laser spot was 0.4 μm. Data were obtained across the 0˗2000 cm^−1^ wavenumber range with a spectral resolution of ~3 cm^−1^ by a cooled CCD detector.

Photomultiplier (PMT) converts optical radiation acquired from the sample into electrical signals. The laser optical scheme is designed to produce a confocal picture of a sample from laser light. Confocal 2D pictures can be formed by scanning the sample and simultaneously recording the intensity of the reflected laser light, recorded with PMT.

### 2.4 Solution chemistry

**FIGURE A5:**
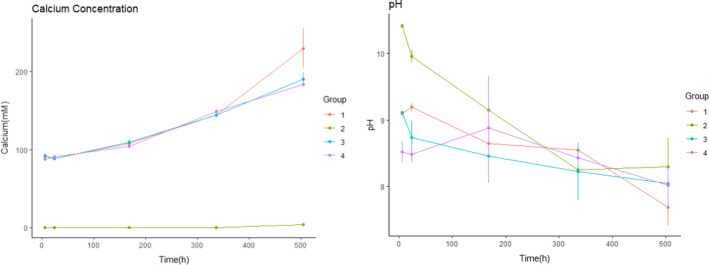
Calcium concentration and pH of the bulk solution throughout the experiment.

The pH of the bulk solution was measured on days 0, 1, 7, 14, and 21 with a Mettler Toledo pH meter (Figure A5). At the same time, a 1 mL bulk solution was filtered through a 0.45 mm cellulose acetate membrane filter to analyze dissolved Ca^2+^ concentration by iCE 3500 Atomic Absorption Spectrometer (ThermoUnicam) equipped with a Deuterium lamp. Other cations, such as K, Na, Mg, Al, Sr, and Ba were detected by Optima 7300 Inductively 658 Coupled Plasma Atomic Emission Spectroscopy. The detection limits for K, Na, Mg, Al, Sr, and Ba were 0.005; 0.005; 0.010; 0.050; 0.0006; and 0.001 µg/mL, respectively.

### 2.5 ANOVA analysis

We used a two‐way mixed effect ANOVA model in STATISTICA to examine the statistical significance of the differences in Cell Coverage among the groups and time intervals studied. In our ANOVA model, the treatment groups were specified as a fixed effect factor and time as a random factor. The *p*‐value was compared against a 5% level of significance to infer whether the differences among the compared means suggest rejection of the null hypothesis (*Ho*). To test the significance of effects in our mixed effect model, we opted for Satterthwaite's method of denominator synthesis, which finds the linear combinations of sources of random variation that serve as appropriate error terms for testing the significance of the respective effect of interest (Satterthwaite, 1946). We also conducted post hoc comparisons to identify the actual sources of statistical significance of our ANOVA results. We used the Bonferroni test to control for an excessively high Type I Error in our analysis.

## 3 RESULTS AND DISCUSSION

### 3.1 Adsorption of cells on mortar surface

**FIGURE A6:**
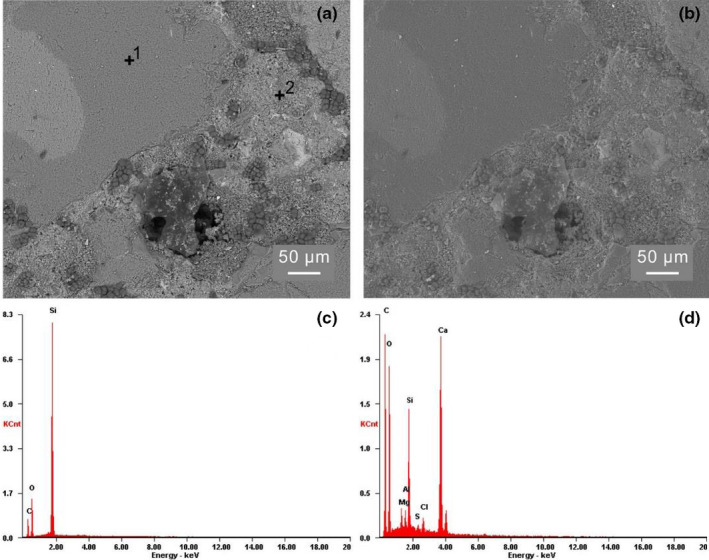
SEM images and EDS spectra on mortar treated with live cells on day 1. (a) Backscattered electron image. (b) Secondary electron image. (c) EDS spectrum corresponds to spot 1 in (a). (d) EDS spectrum corresponds to spot 2 in (a).

All mortars were half immersed in the cell suspension with an equal concentration of cells (10^9^ cells/ml). On cell‐treated mortar surfaces, the majority of the cells adhered to the rough and nanometer‐sized cement paste (white arrows in Figure 2a, b, and c), while few cells attached to the sand grains (black arrows in Figure 2a and b). The differentiation between sand and cement is described in the Appendices (Figure A6). On the total counted area on mortar surfaces, the ratio between cement and sand was close to 1:1 (Figure 2d), and the cell density on the cement paste (counted manually) was 2.1 ± 0.3 × 10^3^ cells/mm^2^, whereas that on sand grains was one order of magnitude lower (Figure 2d). One limitation of the present study is that we assumed the cells form a single layer on the mortar surface, which may result in errors. The observation of the preferential attachment was similar to other studies showing that the density of calcified bacterial cells attached to the quartz grains was 1 order of magnitude lower than those observed on calcitic stone (Ferris et al., 1989; Carlos Rodriguez‐Navarro et al., 2012). In cement structures, the carbonation of portlandite produces calcite, which allows a higher cell attachment (De Muynck et al., 2008). This process will subsequently contribute to a higher cohesion of the bio‐deposition. Electrostatic interactions are one explanation of the bacterium‐mineral adhesion on hydrophilic calcite and sand (Ozkan & Berberoglu, 2013). Bacteria with a high negative surface charge are more easily retained by the positively charged surface (Ozkan & Berberoglu, 2013). On calcitic substrates, the attractive forces easily outweigh repulsion forces; therefore, cells achieved a higher attachment rate on calcites than on sand (Carlos Rodriguez‐Navarro et al., 2012). Surface roughness has also been reported to be an important parameter influencing the adhesion of cells to the hard substrate (Sekar et al., 2004). This attachment preference is significant in the real application of microbial restoration of mortar, since cement paste tends to crack due to shrinkage, leaving its nanometer‐sized surface exposed.

The average initial cell coverage on mortar surfaces, calculated as the area covered by cells divided by the total area of the mortar surface counted, was 8.85 ± 2.18% in group 2, 14.75 ± 3.25% in group 3, and 18.42 ± 2.68% in group 4 analyzed by ImageJ. Cells were all intact, and cell lysis was not observed. ANOVA analysis showed that the difference among the three groups throughout the experiment duration was significant (Table 1). The difference between group 2 and group 3 can be explained by the presence of calcium ions since higher ionic strength enhances the initial attachment of cells to the substrates (Surabhi & Arnepalli, 2020). Increasing ionic strength can even change the interaction between the cells and the substrates from repulsive to attractive (Abu‐Lail & Camesano, 2003). The initial coverage of UV‐killed cells in group 4 was higher than that of living cells in group 3, indicating that the UV pretreatment enhanced the adhesion of cells to substrates.

**TABLE 1:** Two‐way ANOVA analysis and post hoc comparison, based on the Bonferroni test, of cell coverage on mortar in three groups

Groups	Cell coverage ‐ Mean	1	2	3
2	8.49	****		
3	11.69		****	
4	13.92			****

*The F value for the group fixed effect was 26.22, which is associated with a p‐value <0.001. The Bonferroni test suggests that the differences among all three group means are higher than what someone would expect if the null hypothesis held true.

### 3.2 Detachment of cells from mortar surface

**FIGURE 3:**
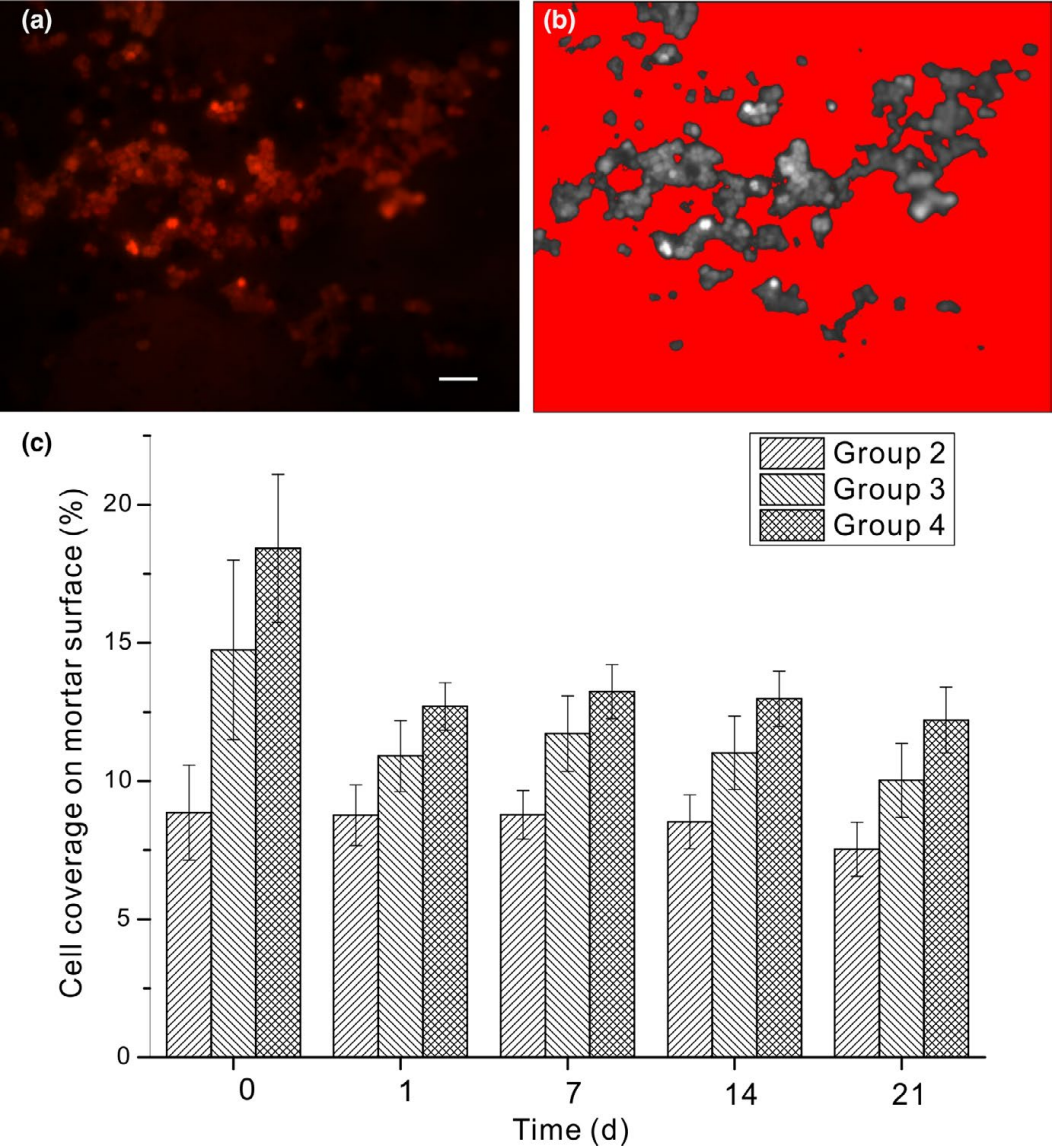
Cell coverage on mortar surfaces of different days and conditions (scale bar is 50 µm)

The density of the cells on the mortar surface in group 3 on day 1 (Figure 2b) was much lower than on day 0 (Figure 2a), indicating that a detachment of cells from the mortar surface occurred within the first day. The cell coverage on mortar surfaces in groups 3 and 4 on day 1 was significantly lower than on day 0 as well (Figure 3c and Table 2), confirming the results observed with SEM. The cell coverage decreased from 14.75 ± 3.25% to 10.90 ± 1.28% in group 3 and from 18.42 ± 2.68% to 12.71 ± 0.86% in group 4 during the first day. After day 1, the cell coverage in groups 3 and 4 kept constant (Figure 3c and Table 2). The cell coverage on mortars in group 2 was about 8.85 ± 2.18% on day 0 and kept constant till the end of the experiment (Figure 3c and Table 2). The difference between the detachment in group 3 and group 2 can be explained by a decrease in ionic strength of the solution in group 3 while that in group 2 remained constant (Surabhi & Arnepalli, 2020). In this study, the concentration of calcium was measured as an indicator of ionic strength. The decrease of the ionic strength was caused by the precipitation of calcium carbonate. A similar process applied to group 4. The detachment percentage for group 3 was 3.85% (or 14.74%–10.90%), and for group 4 was 5.71% (or 18.42%–12.71%). Since the initial attachment percentage was different for group 3 (14.74%) and group 4 (18.42%), the detachment percentage for the two groups cannot be compared directly. Both values were divided by their initial attachment percentage to calculate the percentage of cells detached out of the total cell coverage on the mortar surface at the beginning. The normalized percentage of cells detached in group 3 was 26%, calculated as (14.75–10.90)/14.75, and in group 4 was 31%, calculated as (18.42–12.71)/18.42 (Figure 3). The detachment rate of living cells in group 3 and UV‐killed cells in group 4 was not significantly different. Throughout the experiment, the cell coverage was the highest in group 4 using UV‐killed cells. This indicates that UV‐killed cells may be more effective in real‐world applications than living cells. Nevertheless, further work to shed light on the different ionic strengths, mechanical and water absorption tests needs to be done to reach a firm conclusion.

**TABLE 2:** Two‐way ANOVA analysis and post hoc comparison, based on the Bonferroni test, of the interactive term between the cell coverage on mortar and the time intervals in which values were recorded

Groups	Days	Cell coverage ‐ Mean	1	2	3	4	5	6	7
2	21	7.53	****						
2	14	8.52	****	****					
2	1	8.76	****	****	****				
2	7	8.78	****	****	****				
2	0	8.85	****	****	****				
3	21	10.02		****	****	****			
3	1	10.90		****	****	****	****		
3	14	11.05			****	****	****		
3	7	11.72				****	****		
4	21	12.30				****	****	****	
4	1	12.71					****	****	
4	14	12.93					****	****	
4	7	13.24					****	****	
3	0	14.75						****	
4	0	18.42							****

*The F value for the interactive term between treatment groups (fixed effect) and time (random factor) was 5.89, which is associated with a p‐value <0.001. The Bonferroni test identified the homogeneous groups in which no statistically significant differences exist. Shaded blocks of cells represent the four groups that are distinctly different with minimal overlap in terms of the combinations of treatments and time included. The results showed that group 4 on day 0 was significantly different from all other combinations of days and groups. Although group 3 on day 0 did not display a statistically significant difference with group 4 on days 1 to 21, it was significantly different from all other days in group 3. In comparison, all days of group 2 were classified into a single homogeneous cluster.

Cells were not initially inoculated in the bulk solution, and the expected cell count should have been zero. However, a cell concentration of 10^3^ to 3 × 10^3^ cells/ml was detected in bulk solutions (15 ml) throughout the experiment owing to the detachment from mortar surfaces. Assuming that they were initially attached to the mortar surface (225 mm^2^) as a single layer, the detached cell density would be 66 to 198 cells/mm^2^. Compared to the cell density on mortar surfaces in group 3 on day 0 (Figure 2d), detached cells accounted for about 2.85% to 8.50%. However, the percentage of cells detached in group 3 was 26%, and in group 4 was 31% (Figure 3). The disagreement of the detachment percentage on the mortar surface and in the solution can be explained by the re‐attachment on the other half of the mortar surface that was not immersed in the cell suspension, as observed under SEM. The re‐attachment was more selective on cement paste than on sand grains as well. Further investigations need to be carried out regarding the mechanisms that could reduce the detachment of cells and/or control the re‐attachment sites.

### 3.3 Composition analysis on cell surfaces, mortar, and particles

**FIGURE 4:**
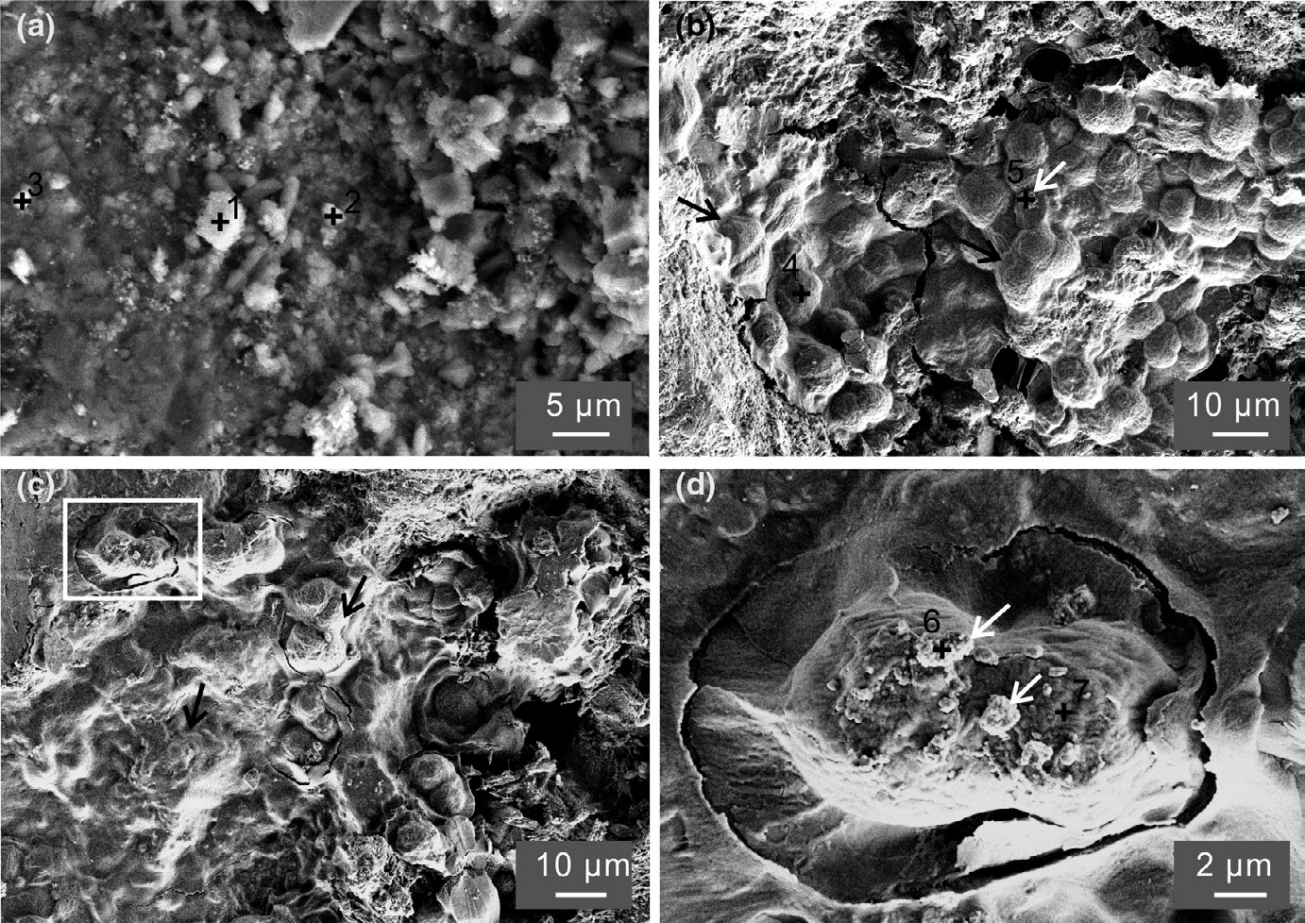
SEM images show (a) particles on the mortar surface‐treated abiotically in group 1; (b) cells (black arrow) attach to the mortar surface in group 3 after 1 day, and few particles (white arrow) adsorbed on cells; (c) EPS (black arrow) around cells in group 4 after 1 day were much thicker than those in group 2, and wrapping the cells; (d) the enlarged area of the white square in (c) showing cells adsorbing particles

**FIGURE A7:**
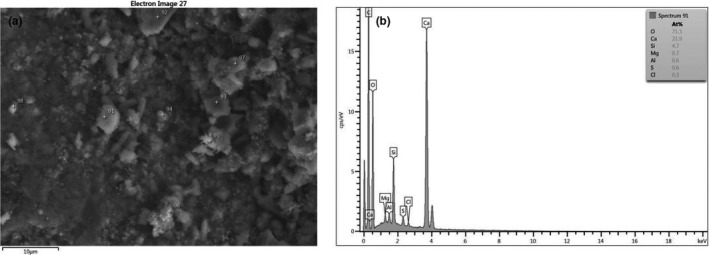
SEM image and EDS on mortar treated abiotically on day 1.

Nanometer‐sized and sub‐micrometer‐sized particles are presented on mortar surfaces in group 1 (Figure 4a and Figure A7) as well as on cells in groups 3 and 4 (white arrows in Figure 4b and d). The EDS on particles in group 1 (black crosses 1 and 2 in Figure 4a) indicates they were a mixture of calcium silicates, calcium magnesium silicates, and calcium aluminates (Table 3). These are typical compositions of cement paste, indicating they were debris from mortar surfaces. A trace amount of Chloride (Cl) from the bulk solution was detected on these particles. In comparison, cells and particles on cells (black crosses 4 and 5 in Figure 4b) in group 3 had a higher content of Cl (Table 3). It was discovered that cyanobacterial cells are passively permeable for Cl, and therefore, they have the tendency to accumulate Cl (Stamatakis et al., 1999). In addition, P (not shown in the table) was detected with an atomic percentage of up to 7.4% on cells (black cross 5 in Figure 4b). The particles on cells having a similar amount of Cl as the cells might be due to the incorporation of organic matter from cells. Some particles (black cross 5 in Figure 4b) did not contain Si or Al, denoting that they were newly formed precipitates. Other particles, having similar amounts of Si and Al as those on mortar surfaces in group 1, might be debris from the mortar that was adsorbed by cells. Cells in group 4 increased their EPS thickness as a stress response to UV exposure (Wingender et al., 1999), but were killed due to prolonged UV treatment. As a result, UV‐killed cells in group 4 were covered by a thicker EPS layer compared to cells in group 3 (Figure 4c). This might be the reason more cell coverage was observed after UV pretreatment (Figure 3c). EPS are found to aid cells in adhering to substrates (Xu et al., 2020). The UV‐killed cells and the particles around them had the highest amount of Cl (black crosses 6 and 7 in Figure 4d, Table 3). The higher amount of Cl might be retained by a higher content of organic matter as a thicker EPS layer was produced. This indicates that EPS and cells have the potential of absorbing chlorine ions, which would otherwise cause corrosion and deterioration of concrete or mortar over time (Achal & Mukherjee, 2015). Similarly, particles on cells could be newly formed precipitates or debris from mortar surfaces.

**TABLE 3:** Composition of particles and cells in group 1 (abiotic condition, sites 1, 2, and 3), group 3 (living cells with calcium, sites 4 and 5), and group 4 (UV‐killed cells with calcium, sites 6 and 7) corresponding to the site numbers in Figure 4

Site	O (at.%)	Ca (at.%)	Si (at.%)	Mg (at.%)	Al (at.%)	S (at.%)	Cl (at.%)	P (at.%)
1	71.3	21.9	4.7	0.7	0.6	0.6	0.3	–
2	77.0	12.1	1.8	0.4	0.6	7.6	0.5	–
3	80.0	16.2	1.7	1.1	0.9	0.2	0	–
4	79.6	14.7	2.1	0.3	0.4	1.0	1.8	0.1
5	74.7	11.0	–	0.4	–	1.6	4.9	7.4
6	70.7	11.4	3.3	3.6	1.2	3.0	5.6	‐
7	70.8	11.4	–	–	–	2.9	14.5	0.3

The following elements are not included in this table: site 6, Fe (at.% = 0.6), Zn (at.% = 0.4) and Cu (at.% = 0.3).

**FIGURE 5:**
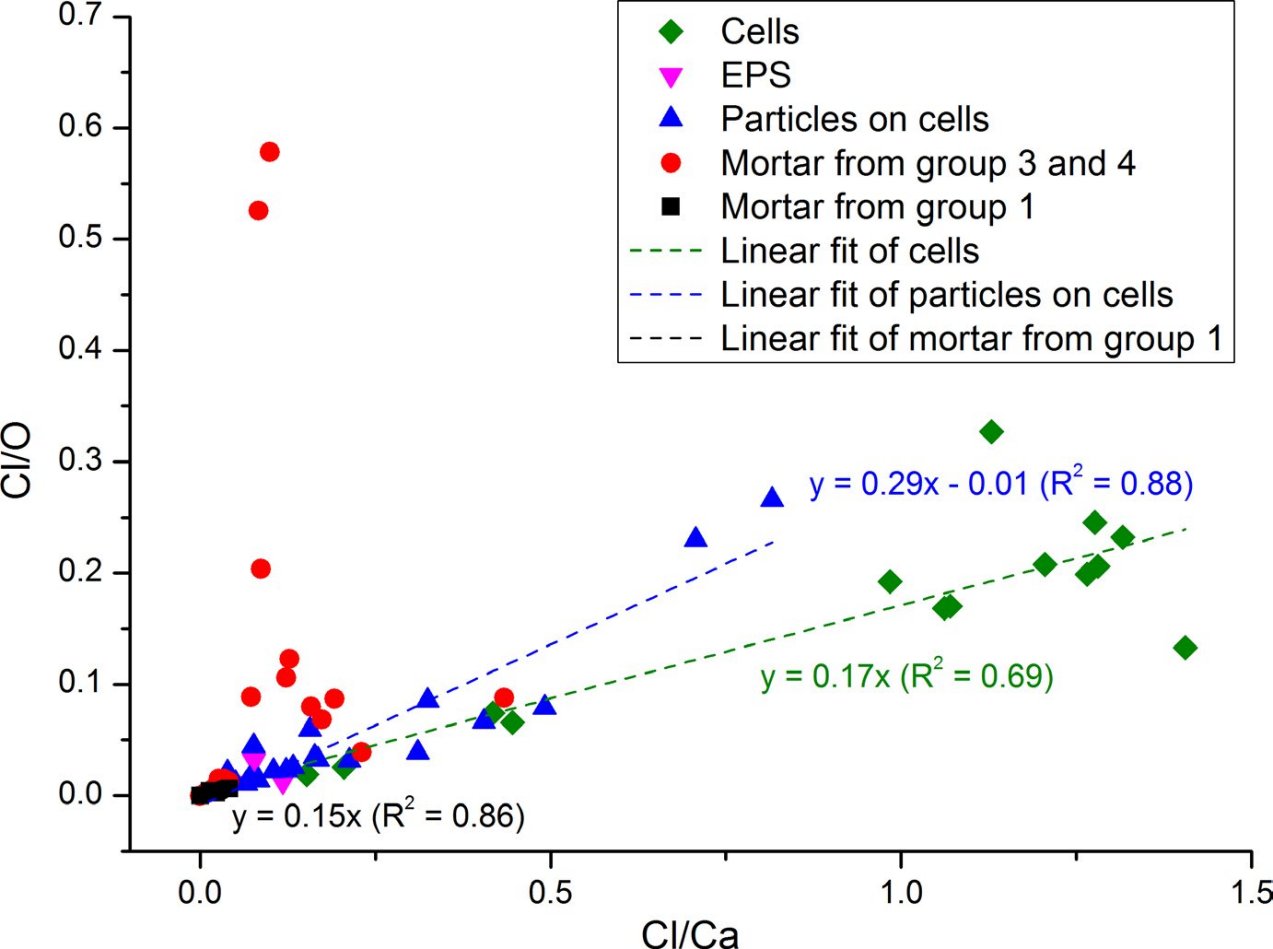
Linear regressions between Cl/Ca and Cl/O on cells, EPS, particles, and mortar surfaces in different groups

The EDS analysis was carried out on 80 different sites on mortar surfaces, cells, EPS, and precipitates. Since the absolute percentage of the elements are not accurate due to carbon coating, we report the ratios between elements for comparison purposes. The ratio of Cl to O and Cl to Ca of particles on cells, mortar surfaces, cells, and EPS were plotted (Figure 5). The ratio of Cl to O and Ca on mortars from group 1 was close to 0, indicating that little or no CaCl_2_ salt precipitated from the bulk solution. A high Cl/O ratio was registered in cells, particles on cells and mortar surfaces pretreated with bacteria in groups 3 and 4, indicating that cells accumulated Cl. Cl/Ca in cells varied between 0.2 and 1.4, suggesting that chloride was incorporated in cells, and other forms of calcium precipitates were deposited on cells as well. Similarly, Cl/Ca in particles on cells varied between 0 and 1.8. Cl/Ca on bacteria‐treated mortars mainly varied between 0.1 and 0.2, possibly due to the debris of EPS covering the surface of the mortar.

In addition, the slope of the linear regression in Figure 5 represents the ratio of Ca and O. In precipitates on cells, this ratio was 0.29, which was close to the ratio of Ca and O in CaCO_3_. The beam with a size of <1 μm, picking up the surrounding signals, contributed to the ratio of Ca and O lower than 0.33. *R* ^2^ of the linear regression between Ca and O on cells was 0.88, which was also statistically significant (*F *= 134.06, *p *= 4.73 × 10^−10^). In this experiment, additional carbon sources besides atmospheric CO_2_ were not provided, so the formation of calcium carbonate confirms that cyanobacteria are capable of using dissolved atmospheric CO_2_ to nucleate CaCO_3_ (Obst et al., 2009). On mortar surfaces in group 1, the Ca/O was around 0.15 with an R^2^ of 0.86 (*F *= 37.42, *p *= 1.73 × 10^−3^). The chemical composition of mortar is very complex, including SiO_2_, Al_2_O_3_, Fe_2_O_3_, CaO, MgO, SO_3_, Na_2_O, and K_2_O (Guler et al., 2020). On mortar surfaces in group 3 and 4, the regression between Ca and O is not significant, with *R* ^2 ^= 0.044, *F *= 1.11, and *p *= 0.30. This inconsistency between the mortar surfaces in the abiotic and biotic experiment can be explained by a mixed signal of mortar compositions and the microbially induced carbonate precipitation. On cells, Ca/O was around 0.17, with *R* ^2^ of 0.69 (*F *= 26.57, *p *= 2.39 × 10^−4^). The lower ratio of Ca/O on cells relative to the precipitates on them can be explained by a higher amount of O in organic molecules in the cells. The lower *R^2^* value is largely driven by two deviating (green) points in Figure 5. One of the two cases displayed an excessively high amount of oxygen while the other point is associated with an extremely high percentage of carbon, which made the concentration of other elements closer to the detection limit, resulting in a less accurate ratio. Another factor that needs to be taken into consideration is the spatial resolution (<1 μm) of the EDS beam. For particles with a much smaller size, the beam picked up the surrounding signals as well.

### 3.4 Calcium carbonates distribution around cells

3.4.1

**FIGURE 6:**
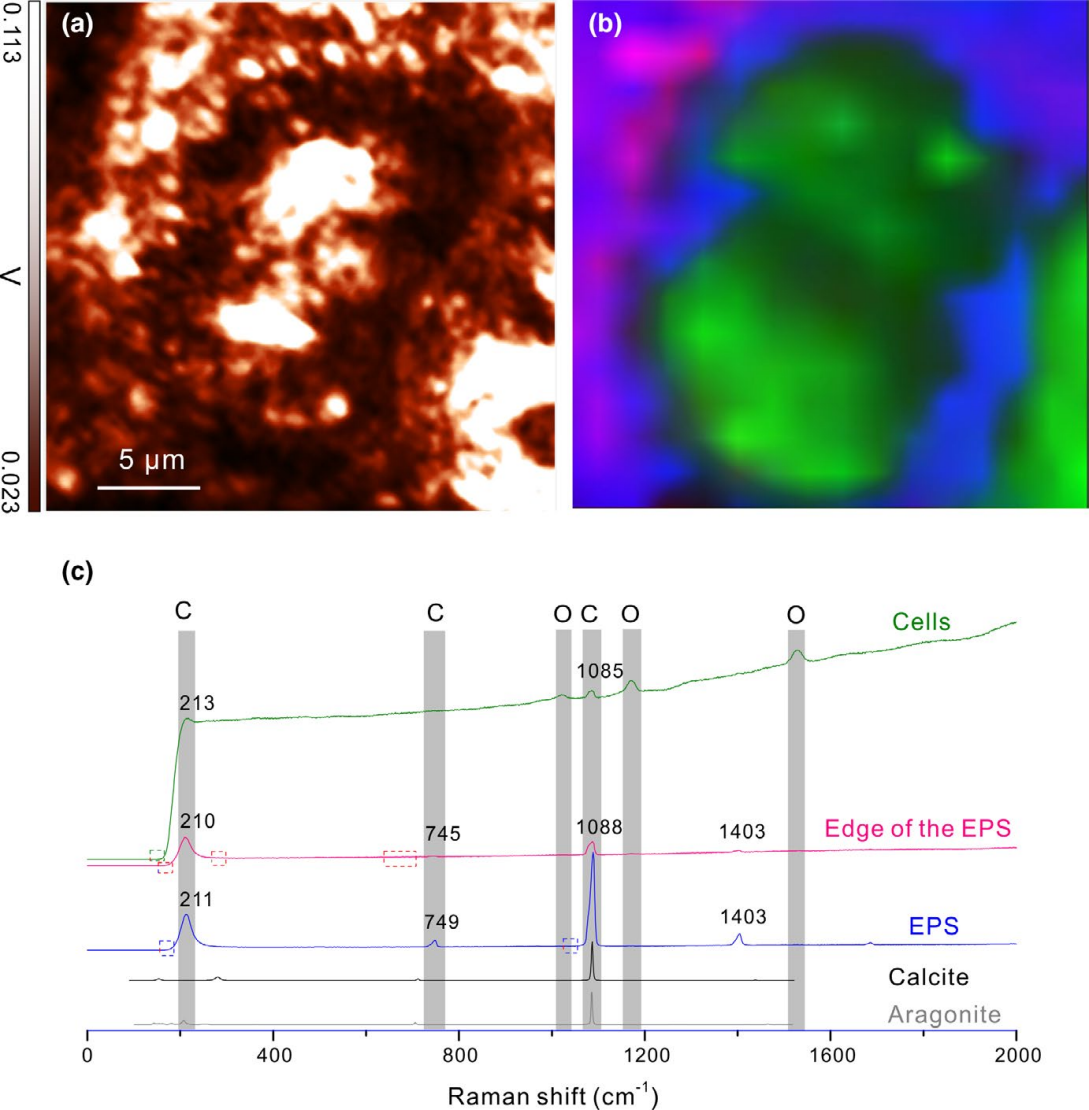
(a) PMT mapping showing a *Gloe*. PCC 73106 setting on the mortar surface. (b) Raman mapping showing green overlapped with cells, blue overlapped with EPS, and pink was on the edge of the EPS. (c) Raman spectra corresponding to the Raman mapping (blue: aragonite and vaterite peaks on EPS, pink: aragonite peaks on the edge of EPS, green: scytonemin and carotenoids peaks as well as aragonite/vaterite peaks on cells)

*Gloe*. PCC 73106 cells with EPS in the middle and right bottom corner of the image square were captured with PMT (Figure 6a). Both cells and EPS were highly reflective due to autofluorescence. The reflection on the left top corner was much lower, corresponding to the surface of the mortar. Raman mapping was collected on the same area (Figure 6b). The green area in the mapping showed peaks at 213, 1088, 1171, and 1527 cm^−1^ (the green curve in Figure 6c), indicating a mixture of carbonates and organic matter (Tables 4 and 5). The green area overlapped with *Gloeocapsa* cells that contain pigments scytonemin and β carotene (Edwards et al., 2005; Edwards et al., 2005). Carotenoids show strong peaks in the 1400 – 1600 cm^−1^ region indicating ν_1_(C=C) stretching vibration (Withnall et al., 2003). The correlation between ν_1_(C=C) and the number of double bonds indicates the chain length of the carotenoids (Merlin, 1985; Withnall et al., 2003). In this study, the peak at 1527 cm^−1^ suggests the presence of β carotene (Kleinteich et al., 2017; de Oliveira et al., 2015). Other signature peaks at 1156 cm^−1^ and 1007 cm^−1^ are very weak, due to the interference of autofluorescence. The peak at 1171 cm^−1^ indicates scytonemin (Edwards et al., 2000), while other peaks at 1590, 1549, 1444, and 1323 cm^−1^ are missing due to impurities. The peaks at 213 and 1088 cm^−1^ indicated aragonite. The blue layer exhibited peaks at 213, 745, and 1088 cm^−1^, revealing that vaterites and aragonites resided in it (Table 4). This blue layer was overlapped with the EPS. The red layer showed peaks on 213 and 1088 cm^−1^, which indicated aragonites. Aragonites were all around the edges of the EPS layer, indicating that the interface of mortar and EPS prefers the formation of aragonites over vaterites. Aragonites and vaterites are polymorphs of calcium carbonates (Tourney & Ngwenya, 2009). Zhang et al. (2018) reported that cells and their organic macromolecules (e.g., exopolysaccharides or proteins) control the morphology and mineralogy of the calcium carbonates (Zhang et al., 2018). On the left top corner of the image square, it was a light blue layer, indicating a much lower amount of vaterites and aragonites on mortar surface than on EPS. The formation of vaterites and aragonites on the mortar surface was due to the carbonation of portlandite (Martinez‐Ramirez et al., 2003). In addition, small pieces of EPS might be released and deposited in the neighborhood of vaterites and aragonites. Whereas their formation on EPS was due to the calcium carbonate precipitation in the microenvironment where calcium ions were concentrated, CO_2_ was dissolved in the solution and transformed to HCO_3_^−^. Carbonates on cells were aragonites only, suggesting that cells attracted a higher concentration of Mg^2+^ than the EPS in the microenvironment as Mg/Ca ratio is a governing factor of CaCO_3_ polymorphs (Davis et al., 2000). Both cells and EPS were able to nucleate carbonates in the absence of bicarbonates or organic carbon source. The nucleation is essential for the subsequent carbonate precipitation, serving as the template for crystal growth, which in turn could lead to CO_2_ sequestration as well.

**TABLE 4:** Raman peak identification for carbonate polymorphs from literature and this study

Carbonate polymorphs	Lattice mode	ν_4_ in‐plane bending	ν_1_ symmetric stretching	References
Calcite	150, 281	711	1084	(Sevcik & Macova, 2018)
	155, 282	711	1085	(Nehrke et al., 2012)
	279	709	1082	(Liang et al., 2013)
Aragonite	150, 205	701	1084	(Sevcik & Macova, 2018)
	143, 153, 162, 181, 207, 249, 261, 275, 284	701, 706, 717	1085	(Wehrmeister et al., 2010)
	155, 206	705	1085	(Nehrke et al., 2012)
	208	700	1083	(Paulo & Dittrich, 2013)
	155 (w.) 213	701 (w.), 707 (w.)	1085	This study
Vaterite	150, 205, 281	750	1084	(Sevcik & Macova, 2018)
	210, 280, 300	740, 750	1076, 1090	(Martinez‐Ramirez et al., 2003)
	105, 114, 267, 300	740, 750	1075, 1090	(Nehrke et al., 2012)
	150 (w.), 280 (w.)	745	1088	This study

Abbreviation: w., weak.

**TABLE 5:** Raman peak identification for β carotene

	ν _1_(C=C)	ν _2_(C–C)	ρ(C–CH_3_)	References
β Carotene	1525	1158	1005	(Merlin, 1985)
	1515 1520	1155 1157	1000 1004	(de Oliveira et al., 2015)
	1529	1159	1006	(Kleinteich et al., 2017)
	1518	1156	1003	(Edwards, Moody, Villar, et al., 2005)
	1519	1155	1005	(Withnall et al., 2003)
β Carotene	1527	1156 (w.)	1007 (w.)	This Study

Abbreviation: w., weak.

On UV‐killed samples, Raman spectra were carried out on individual points and provided similar results to those for living cells. This resemblance might be due to the same functional groups residing in UV‐killed and living cells that induced calcium carbonate precipitation. This leads to the conclusion that functional groups in EPS and cells were the main driving factor for carbonate precipitation. Similar conclusions about the crucial role of cell surfaces in calcium carbonate nucleation were proposed for other cyanobacterial strains *Synechococcus elongatus* (Obst et al., 2009). On the living cells without calcium ions in group 2, the peaks on cells indicated scytonemin and carotenoids, but not carbonates, denoting that calcium carbonates formed on cells in groups 3 and 4 used calcium ions in solution rather in mortar. At least 20 Raman spectra were collected from each group. In group 3, 30% of the spectra showed carbonate peaks, while in group 4, 35% of the spectra showed carbonate peaks. This finding is important for real‐world applications since degradation of the mortar may occur if the cells use the calcium from the mortar.

This study focuses on the underlying processes including the attachment of cells and early stages of carbonate precipitation on mortar. It is the first time that the percentage of microbial attachment on mortar is reported quantitatively. Using regression analysis in interpreting the composition of nanometer‐ to micrometer‐sized particles is useful for investigating early‐stage precipitation and is transferable to similar research. This study is the first step toward the real‐world application of CCP in mortar/concrete restoration. The capability of *Gloe*. *PCC 73106* sequestrating CO_2_ in mortar restoration has significant environmental implications. In 2019, the global concrete repair was estimated at 1.7 × 10^7^ metric tons, based on the concrete repair market at USD 2.1 billion, and the average cement price at USD 123.5 per metric ton. Producing a ton of cement generates nearly a ton of CO_2_. If 1% of the repairing volume is replaced by CCP, it reduces the CO_2_ emission by 1.7 × 10^5^ metric tons per year, while sequestrates 7.5 × 10^4^ metric tons of CO_2_. In addition, cyanobacteria convert CO_2_ to biomass during the incubation stage. Another possible application of CCP is to reduce the subsurface leakage of geologically sequestered CO_2_ where the carbon source is readily available, and the low viscosity aqueous solution makes it more advantageous to reach fractures in‐depth compare to cementitious grouts (Cuthbert et al., 2013; Phillips et al., 2016).

## 4 CONCLUSIONS

We investigated the attachment of cyanobacteria *Gloe*. PCC 73106 on mortar surfaces and their carbonate precipitation in the absence of inorganic or organic carbon sources. Based on the following summary, this study provided evidence that the attachment of *Gloe*. PCC 73106 cells on mortar surfaces was enhanced by the UV pretreatment and carbonate precipitation using dissolved atmospheric CO_2_ occurred in the presence of cells and EPS.

1. The initial coverages of living and UV‐killed cells on mortar surfaces were 14.75% and 18.42%, respectively. The UV pretreatment on cells helped them to produce more EPS, which in turn enhanced the cell attachment on mortar.
2. Both living and UV‐killed cells were preferably attached to cement paste than to sand grains, and the difference was one order of magnitude.
3. The detachment of living and UV‐killed cells on the first day was the highest at 26–31% and was close to 0 during the rest of the experiments.
4. In the absence of additional inorganic and organic carbon sources, both living and UV‐killed cells were able to promote carbonate precipitation using atmospheric CO_2_ that was dissolved in water as HCO_3_^−^. The carbonate signal on cells or EPS was much higher than on mortar surfaces.
5. By decreasing the environmental impacts including mitigating the greenhouse effect, the restoration of mortar by CCP is a sustainable application.

## 5 ETHICS STATEMENT

None required.

## Conflict of Interest

None declared.

## AUTHOR CONTRIBUTIONS

**Tingting Zhu:** Conceptualization (equal); Data curation (equal); Formal analysis (equal); Methodology (equal); Writing‐original draft (equal). **Mohamed Merroun:** Methodology (equal); Writing‐review & editing (equal). **George Arhonditsis:** Methodology (equal); Writing‐review & editing (equal). **Maria Dittrich:** Conceptualization (equal); Funding acquisition (equal); Methodology (equal); Writing‐review & editing (equal).

## Data Availability

All data are provided in full in this paper except for the cell coverage summary, which is available in the figshare repository at https://doi.org/10.6084/m9.figshare.16660429.

## APPENDIX 1

### Additional Methods

#### Autofluorescence and viability test

Although a chlorophyll fluorescence filter set (excitation: 435/40nm; beam splitter: 510 nm; and emission: 515 nm) can be used to analyze the viability of unicellular cyanobacteria (Schulze et al., 2011), it is not applicable for this study due to two facts. First, the concrete exhibits strong green fluorescence that can interfere with the green channel (Figure A1). Second, the cells in the viability analysis are heat‐treated (Schulze et al., 2011), while in our study was UV treated. Studies show that the UV treatment does not affect or may slightly increase the chlorophyll content (Ehling‐Schulz et al., 1997; Rumyantsev et al., 2017). The destruction of the autofluorescence compounds was a result of thermal denaturation (Sheng et al., 2011). In this study, the UV‐killed cells were reinoculated in BG‐11 medium in triplicates, and none of them showed the growth of cells. Neu et al. (2004) detected autofluorescence of cyanobacteria using a filter set (excitation 567 nm, emission 600 +/‐ 30 nm, channel red), and our study adopted a similar wavelength using a TRITC filter set (exciter, 546 nm, emitter, 575–640 nm, and dichroic beam splitter, 560 nm). The empirical experience shows that cells exhibit autofluorescence in both blue and red channels (Figure A2). However, since the concrete surface showed scattered blue fluorescence (Figure A1), a red channel was used to inspect cyanobacteria autofluorescence. Both living and UV‐killed cells exhibit autofluorescence in the red channel (Figure A3). The intensity of autofluorescence was preserved for at least 7 days under our experimental conditions (Figure A4). This intensity slightly decreased after 14 days but did not impact the coverage estimation with the selected threshold.

## APPENDIX 2

### ADDITIONAL RESULTS

#### Solution chemistry

The calcium concentrations in groups 1, 3, and 4 decreased to 91.26 mM, 92.04 mM, and 87.95 mM, respectively, after 6 hours on day 0 (Figure A5), due to high pH caused by the concrete. The pH of the bulk solution in group 2 (concrete cubes immersed in 15 ml deionized water without calcium) was 10.41. The pH in other groups decreased on day 0 due to the precipitation of calcium carbonates. The pH decreased to 9.10 in group 1 (the abiotic condition with calcium) and 9.11 in group 3 (fresh cells with calcium). A larger decrease in pH to 8.52 was observed in group 4 (UV‐killed cells with calcium), as a result of a larger amount of calcium carbonate precipitation. As the bulk solution absorbed the CO_2_ from the atmosphere, the pH of the bulk solution decreased. As the pH decreased, the precipitated calcium carbonate became unstable, and the calcium concentration in the bulk solution increased. By the end of the experiment (day 21), the calcium concentration in group 1 (the abiotic condition) was significantly higher than in groups 3 (with fresh cells) and 4 (with UV‐killed cells). Since the calcium was not added in group 2, no calcium precipitation was observed, and the pH was the highest at the beginning. The changes were monitored in the bulk solution at the macroscopic scale, while the microenvironment can be very different around the interface between the mortar and cells. Other cations were below the detection limits.

#### Sand and cement differentiation

The SEM images show the morphological differences, and the EDS show the composition differences between sand and cement (Figure A6). The sand (curser 2 in Figure A6b) is composed of Si and O (Figure A6c), while the cement (curser 7 in Figure A6b) is made up of Ca, Si, Al, Mg, and O (Figure A6d). The sand is much smoother than the cement, while the cement shows roughness at the nanometer scale.
